# UAV-Based Thermal Inversion for Canopy Temperature Retrieval and Precision Irrigation

**DOI:** 10.3390/s26165023

**Published:** 2026-08-07

**Authors:** Haoming Li, Wei Li, Chenchen Liu, Leilei Ji, Zhenbo Liu, Ramesh K. Agarwal

**Affiliations:** 1 National Research Center of Pumps, Jiangsu University, Zhenjiang 212013, China; 2 Department of Mechanical Engineering & Materials Science, Washington University in St. Louis, St. Louis, MO 63130, USA; rka@wustl.edu

**Keywords:** UAV, thermal infrared inversion, canopy temperature, crop water stress index, precision irrigation, stomatal conductance

## Abstract

Accurate assessment of crop water status is critical for precision irrigation and sustainable water management in agriculture. This study develops a UAV-based thermal infrared inversion framework for high-resolution canopy temperature retrieval and irrigation decision support in tea plantations. The proposed approach integrates multi-frame image mosaicking, threshold-based canopy extraction, and a gray–temperature calibration model to generate spatially continuous canopy temperature maps. Crop water stress was quantified using the Crop Water Stress Index (CWSI), and its reliability was further evaluated by analyzing its relationship with stomatal conductance. The framework further estimates soil moisture status and irrigation requirements based on a threshold-based irrigation strategy. The results show that the linear gray-temperature calibration model achieved a maximum absolute error of less than 0.3 °C and that the calculated CWSI and estimated irrigation requirement were strongly correlated with measured stomatal conductance, with *R*^2^ up to 0.91. The proposed method provides a practical technical workflow from UAV thermal imagery acquisition to canopy temperature retrieval and quantitative irrigation decision-making, demonstrating its potential for precision irrigation management in tea plantations.

## 1. Introduction

Agriculture accounts for approximately 70% of global freshwater withdrawals, making it the dominant sector affecting water resource sustainability [1,2]. Under the combined pressures of climate change, increasing evaporative demand, and population growth, improving agricultural water use efficiency has become a central challenge for global food security [3]. Precision irrigation, which relies on the accurate and timely assessment of crop water status, is widely regarded as an effective approach to address this challenge.

Among various plant-based indicators, canopy temperature has been widely used as a sensitive indicator of crop water stress, as it reflects the coupled effects of transpiration, stomatal regulation, and energy exchange within the soil–plant–atmosphere continuum [4,5,6]. Building on this principle, the Crop Water Stress Index (CWSI) provides a normalized method for quantifying water deficit by relating canopy-air temperature differences to environmental baselines [7]. Numerous studies have validated the effectiveness of CWSI across different crops. For instance, Rud et al. utilized canopy temperature histograms to derive CWSI for potato under deficit irrigation conditions [8], while Park et al. reported strong correlations between UAV-derived CWSI and stem water potential in orchard systems [9]. Similarly, Moran et al. proposed the Water Deficit Index (WDI) by integrating thermal and spectral information for more comprehensive stress evaluation [10]. However, most existing thermal stress indices are mainly used for water-stress diagnosis or treatment comparison, and their direct use for quantitative irrigation decision-making remains limited.

Recent developments in unmanned aerial vehicle (UAV) platforms equipped with thermal infrared (TIR) sensors have significantly improved the spatial and temporal resolution of canopy temperature monitoring [11,12,13,14]. UAV-based thermal imaging enables the rapid acquisition of high-resolution temperature information, overcoming the spatial limitations of traditional ground-based measurements. This technology has been successfully applied to assess crop water status in a wide range of agricultural systems, including cotton [15], wheat [16,17,18], tea plantations [19], and vineyards [20]. Moreover, UAV-derived canopy temperature has been shown to be strongly correlated with transpiration and evapotranspiration dynamics, providing a reliable basis for irrigation scheduling [21,22].

However, despite its considerable potential, accurate canopy temperature retrieval from UAV-TIR data remains technically challenging [23]. One critical issue is radiometric calibration and environmental correction. Aragon et al. demonstrated that improper calibration of uncooled thermal sensors can introduce errors exceeding 5 °C, whereas pixel-wise correction significantly improves accuracy [24]. Wan et al. further highlighted the influence of flight altitude, ambient temperature, and sensor drift on thermal measurements [25], while Metsu et al. emphasized the necessity of atmospheric and emissivity corrections for reliable land surface temperature retrieval [26]. These findings indicate that raw thermal imagery cannot be directly used for quantitative canopy temperature retrieval without appropriate preprocessing and calibration.

From the perspective of canopy temperature retrieval, current approaches include physics-based correction, mixed-pixel decomposition, and empirical calibration methods. Spectral unmixing techniques have been developed to separate mixed pixels and retrieve pure canopy temperature, particularly in heterogeneous agricultural scenes [27]. In recent years, multi-source UAV observations have also been used to improve crop water-stress assessment by combining thermal, spectral, and meteorological information [28,29,30]. However, the practical application of these approaches in tea plantations remains constrained by complex canopy structure, soil background interference, limited field samples, and rapidly changing environmental conditions. Therefore, a practical and interpretable temperature retrieval method that can reduce mixed-pixel effects and support field-scale irrigation decision-making is still needed.

In parallel, image processing efficiency remains another major bottleneck. Current workflows heavily rely on commercial photogrammetry software, such as Pix4D and ENVI, which can be computationally expensive and less transparent for algorithm-level analysis [31,32]. Feature-based image registration algorithms, such as SIFT and SURF, are widely adopted due to their robustness to scale and illumination variations [33,34]. The SURF algorithm, in particular, offers a favorable balance between computational efficiency and matching accuracy [35]. However, when processing UAV image sequences with high overlap, traditional pairwise matching strategies may suffer from cumulative errors and reduced efficiency [36]. Additionally, soil background interference introduces significant uncertainty in canopy temperature estimation. Studies have shown that failure to properly isolate vegetation pixels can lead to biased canopy temperature extraction and consequently distort CWSI calculation [37,38]. Therefore, an efficient multi-frame registration strategy and a canopy-focused temperature retrieval procedure are both necessary for reliable UAV-based thermal inversion in row-structured tea plantations.

Beyond temperature retrieval, another key limitation is the weak coupling between UAV-derived thermal information and irrigation decision-making. Although UAV-derived CWSI has been successfully used to differentiate irrigation treatments and monitor crop water status [14,20], translating water-stress indicators into quantitative irrigation amounts remains challenging. Some studies have attempted to bridge this gap by integrating canopy temperature with evapotranspiration models or machine learning-based decision frameworks [21,27], yet a unified and automated pipeline from thermal data acquisition to irrigation decision-making is still lacking.

To address these challenges, this study proposes a UAV-based thermal infrared inversion and irrigation decision-making method for tea plantation systems. Specifically, an improved multi-frame SURF-based image mosaicking algorithm is developed for UAV thermal infrared images. Different from conventional pairwise image stitching, the proposed method performs SURF feature extraction and matching between adjacent frames, estimates projective transformations, cumulatively updates transformation matrices across the image sequence, and introduces center-frame correction to reduce cumulative registration errors. A canopy-focused threshold segmentation method is then employed to eliminate soil background interference before temperature inversion and extract canopy temperature more reliably. Furthermore, the inverted canopy temperature is coupled with a canopy-temperature-driven irrigation decision algorithm that integrates CWSI-based crop water stress estimation, meteorological parameters, soil water status, and irrigation system parameters. The proposed approach aims to bridge the gap between low-level thermal image processing and high-level irrigation management, providing both methodological innovation and practical applicability for precision agriculture, particularly in tea plantation systems.

The main contributions of this study are summarized as follows. First, an improved multi-frame SURF-based mosaicking algorithm is proposed to improve the spatial consistency of UAV thermal mosaics generated from highly overlapped images. Second, a canopy-focused temperature retrieval strategy is established by removing soil background pixels before temperature inversion, thereby reducing the influence of mixed canopy–soil pixels on plot-scale canopy temperature estimation. Third, a canopy-temperature-driven irrigation decision algorithm is developed to translate UAV thermal remote-sensing observations into quantitative irrigation amount recommendations. These contributions enable a more reliable transition from UAV thermal image processing to field-scale irrigation decision-making in tea plantation systems.

## 2. Material and Methods

### 2.1. Study Area and UAV Data Acquisition

#### 2.1.1. Experimental Site

**Figure 1:**
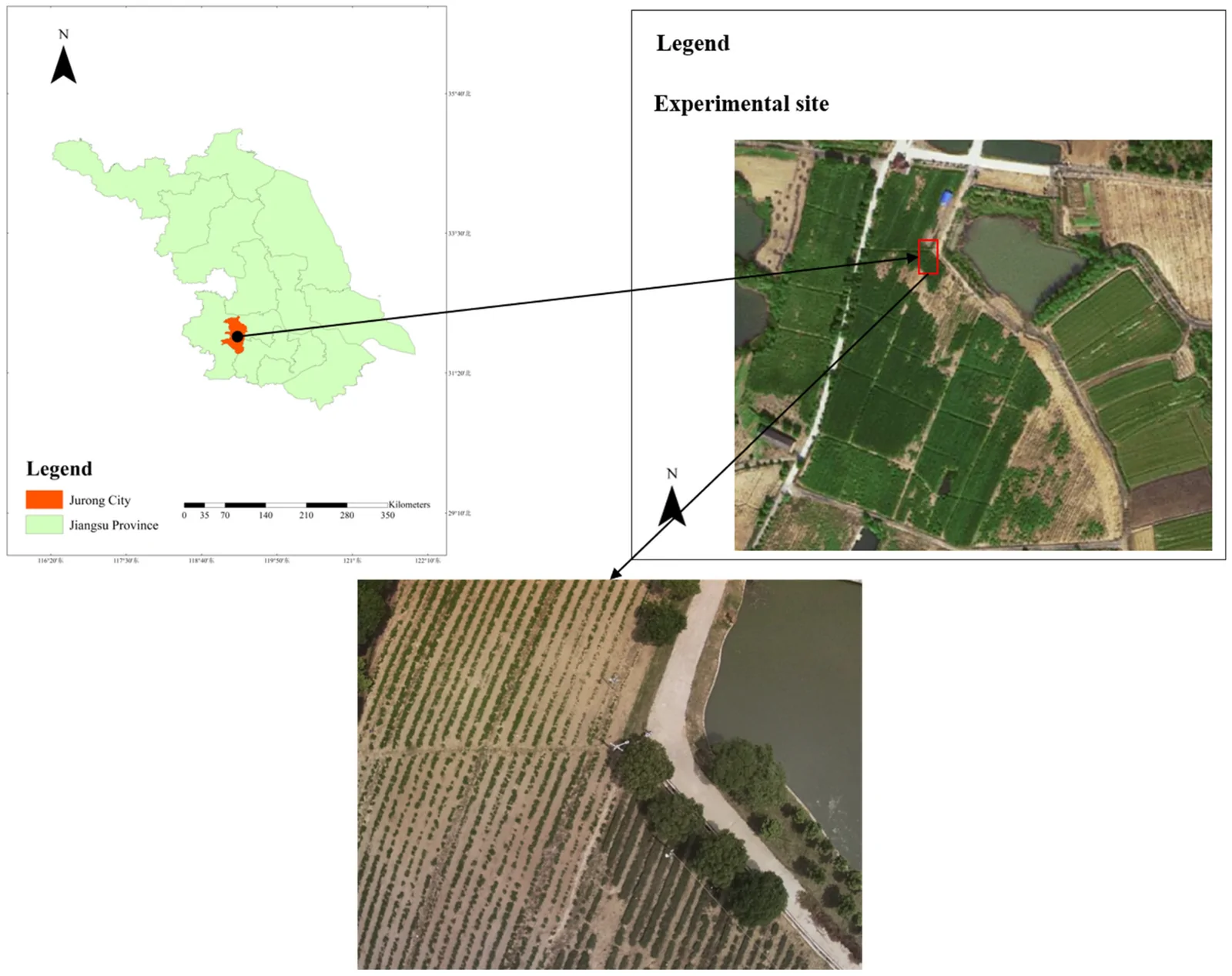
Study map of the experimental site.

**Figure 2:**
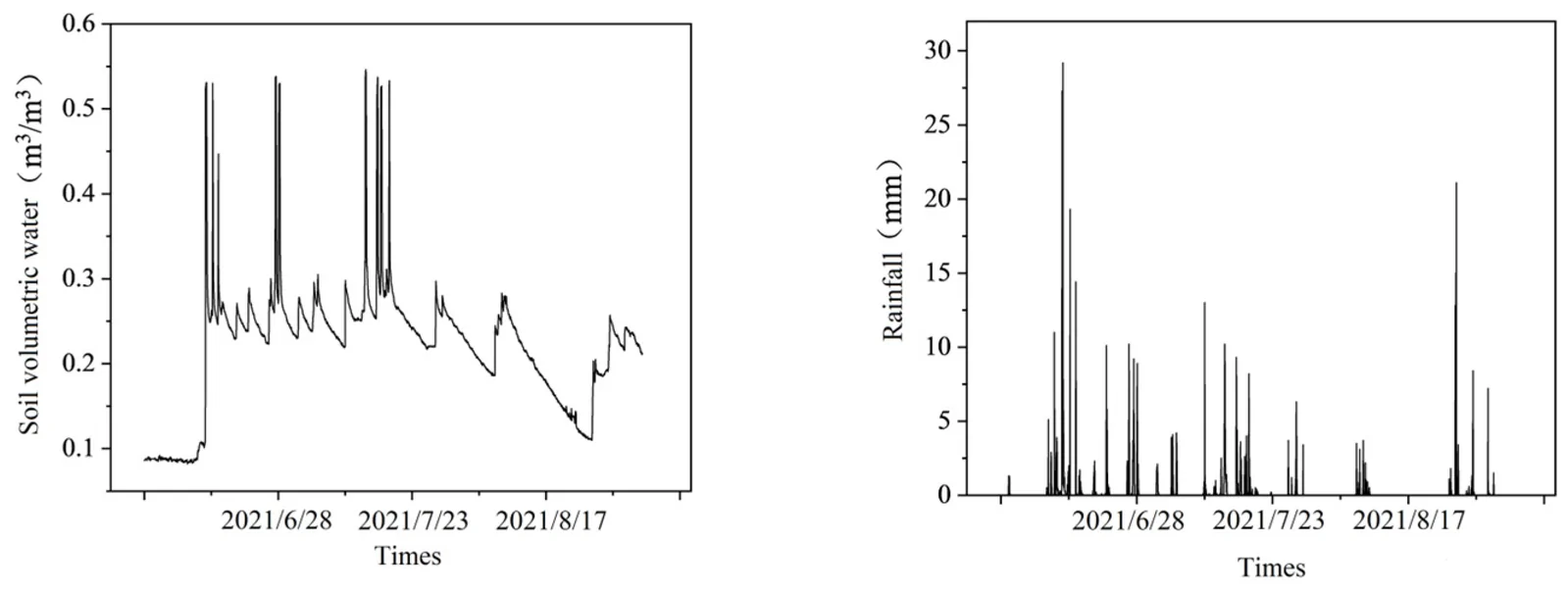
Meteorological and soil data.

The experimental study was conducted in a commercial tea plantation located in Jurong City, Jiangsu Province, eastern China (32°1′00″ N, 119°4′00″ E). The region is characterized by a typical subtropical monsoon climate, with pronounced seasonal variability in temperature and precipitation. During the growing season, ambient temperatures typically range from 25 °C to 40 °C, providing favorable conditions for tea cultivation. The study area is planted with Maojian green tea, with a relatively uniform canopy structure. The canopy height ranges from 0.7 m to 0.8 m, with an average value of approximately 0.75 m, which is suitable for UAV-based high-resolution thermal observation. The soil is classified as silty loam, exhibiting moderate water retention capacity, with a field capacity of approximately 25%, a wilting point of 12%, and a capillary break moisture content of 17.5%. The soil pH is approximately 5.0, which is consistent with optimal conditions for tea growth. To characterize the environmental conditions during the experiment, meteorological variables (air temperature, humidity, solar radiation) and soil moisture were continuously monitored. These parameters are essential for subsequent canopy temperature normalization and CWSI calculation. An overview of the experimental site is shown in Figure 1, while the local meteorological and soil conditions are presented in Figure 2.

#### 2.1.2. Experiment Design

To comprehensively collect remote sensing data, flight missions were conducted on 2 September at three different intervals: 09:00, 11:00, and 13:00. The UAVs were operated at relative flight altitudes of 25 m, 40 m, and 60 m. During the flight missions, wind speed was measured synchronously by the in-field weather station and was 0.97 m/s, 1.36 m/s, and 1.53 m/s at 09:00, 11:00, and 13:00, respectively. Cloud cover was not quantitatively recorded during the experiment; however, field observations confirmed no rainfall or noticeable cloud-shading events during the flights. Two types of DJI drones were utilized: a Phantom 4 RTK (DJI Inc., Shenzhen, China) equipped with an RGB camera (DJI Inc., Shenzhen, China), and a Matrice 300 RTK (M300 RTK, DJI Inc., Shenzhen, China) equipped with a Zenmuse XT2 thermal imaging camera (DJI Inc., Shenzhen, China) to capture RGB and infrared thermal images, respectively. A DJI A3 flight controller (DJI Inc., Shenzhen, China) was used to manage the drone operations. The detailed specifications of the thermal and RGB cameras are summarized in Table 1.

**Figure 3:**
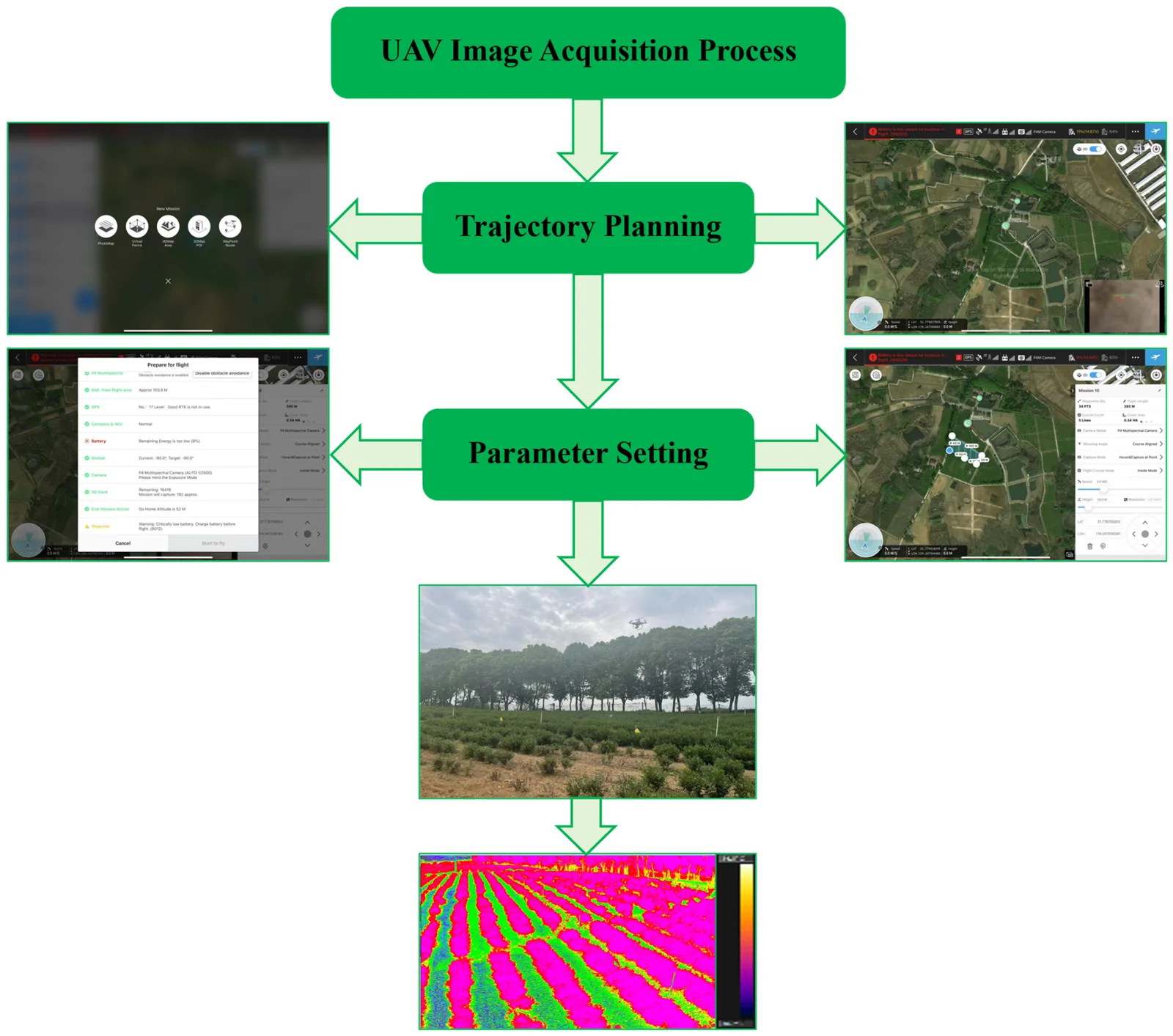
Upload shooting task process.

**Figure 4:**
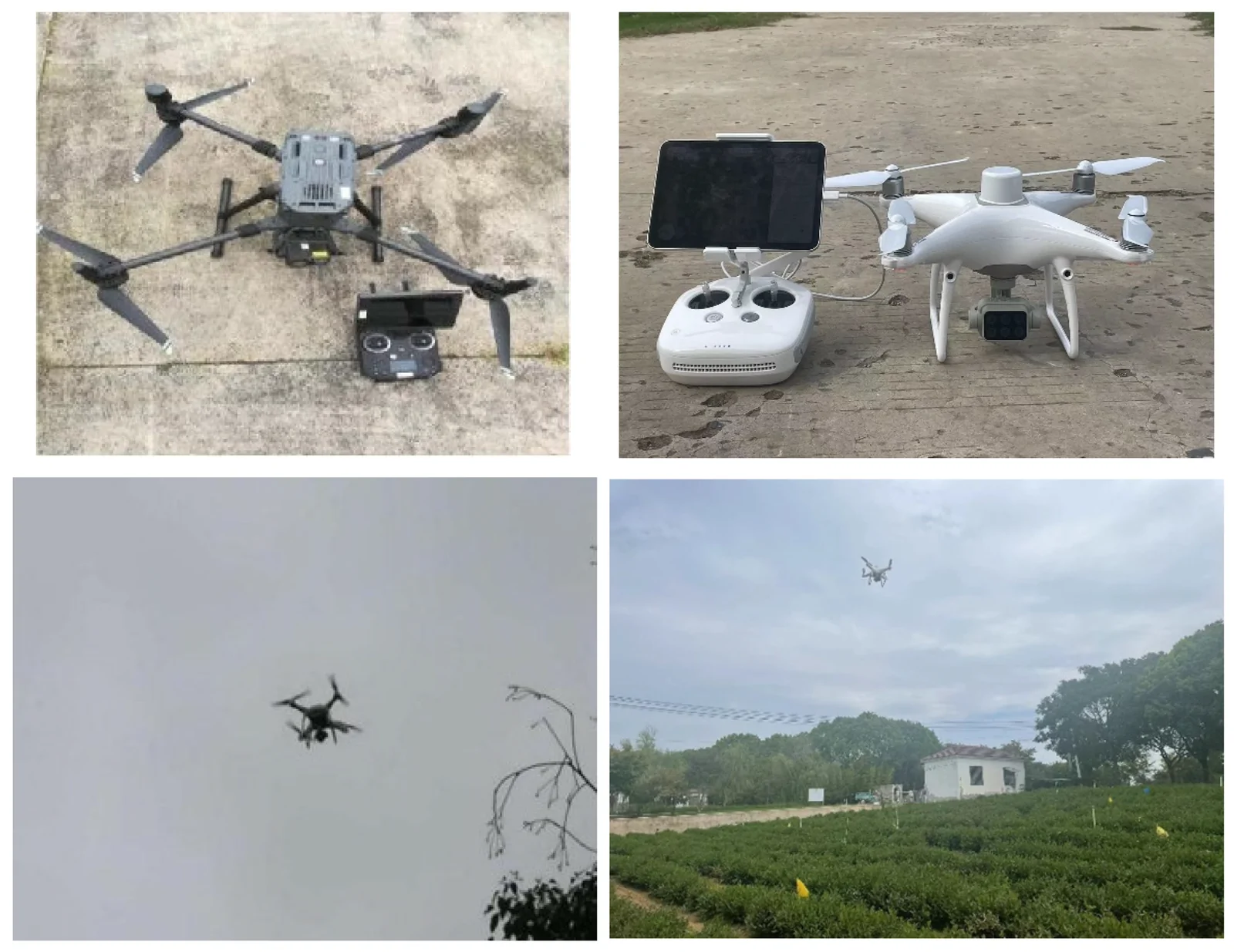
UAV physical and flight operation.

During the flight missions, the sensors were periodically triggered based on pre-programmed shooting tasks transmitted to the UAV platform via the ground control station (Figure 3). In automatic flight mode, a high-precision GPS module (u-blox M8T) was integrated to ensure superior georeferencing accuracy. Each flight was executed at a constant speed of 3 m/s at the designated altitudes to ensure sufficient image overlap. Each captured image (saved in JPEG format) automatically recorded the spatial coordinates of the shooting location. Figure 4 illustrates the physical UAV platforms and the live flight operations.

Synchronously with the UAV flights, in situ leaf temperature measurements were collected using a handheld infrared thermometer for calibration and validation of UAV-derived canopy temperature. A total of 50 ground leaf temperature measurements were obtained during the flight period, among which 40 samples were used for gray–temperature model fitting and 10 samples were used for independent validation. The sampling points were distributed across the experimental tea plantation to cover different canopy temperature conditions and to avoid field-edge effects. For each sampling point, leaves from the upper sunlit canopy were selected because this canopy layer directly corresponds to the surface observed by the UAV thermal sensor. The geographic positions of the sampling points were matched with the processed thermal orthomosaic. To reduce the mismatch between point-scale measurements and image pixels, the mean thermal value within a small region of interest around each sampling point was extracted and used for comparison with the corresponding ground measurement. Following the completion of the flight missions, actual plant heights were physically measured. Ultimately, the collected remote sensing images were processed to accurately extract canopy temperature data for further validation.

### 2.2. Thermal Image Processing Framework

**Figure 5:**
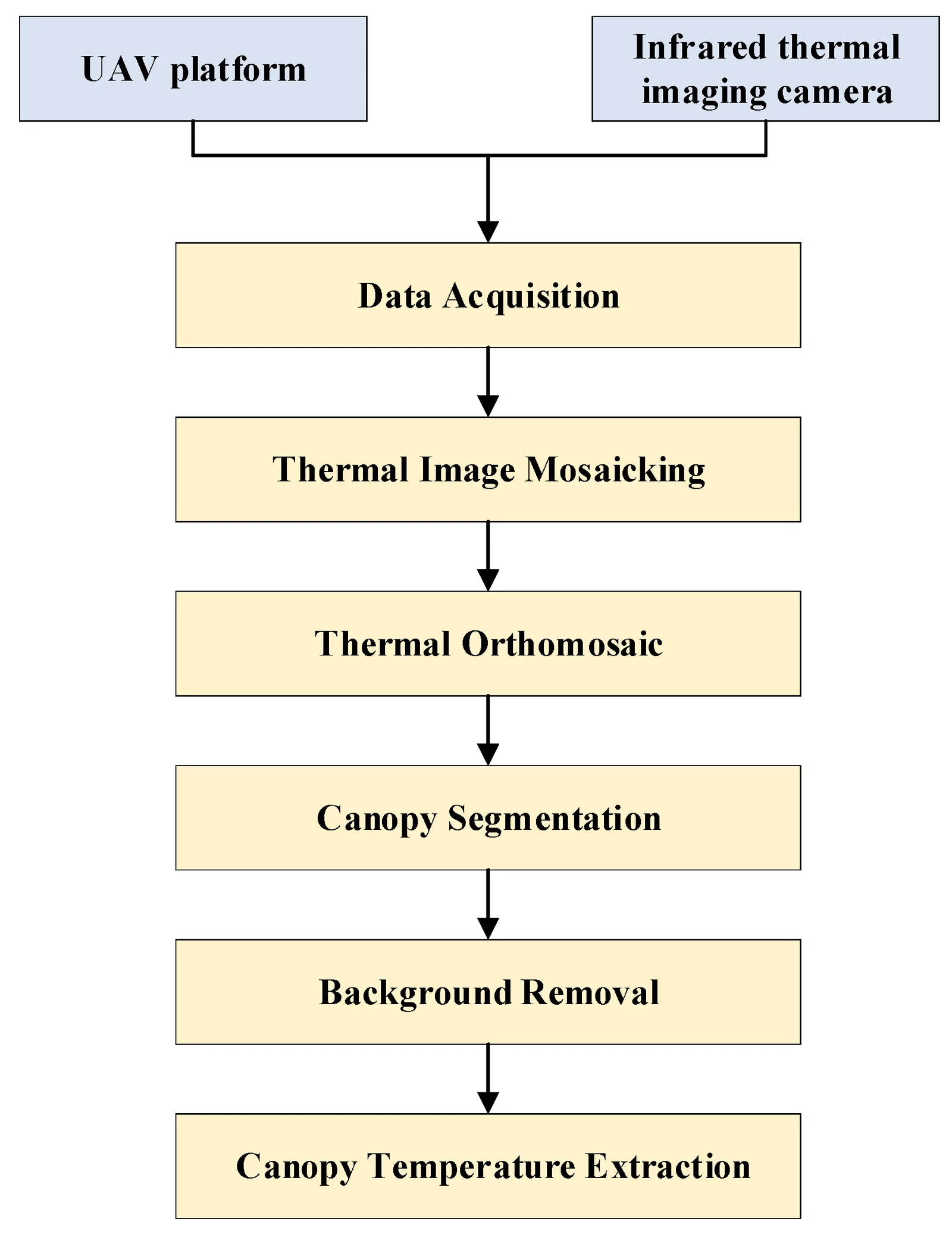
Workflow of the thermal infrared image processing framework for canopy temperature extraction.

An integrated processing framework was established to enable accurate retrieval of canopy temperature from UAV-based thermal infrared imagery. The framework consists of three sequential modules: multi-frame image mosaicking, canopy extraction with background removal, and temperature retrieval. As illustrated in Figure 5, the workflow begins with UAV-based data acquisition and proceeds through geometric alignment and radiometric processing to produce spatially continuous canopy temperature data.

The primary objectives of this framework are: (i) to generate a geometrically consistent thermal orthomosaic from highly overlapped UAV images, (ii) to eliminate soil background interference and isolate canopy pixels, and (iii) to establish a quantitative relationship between image gray levels and physical temperature values.

#### 2.2.1. Multi-Frame Image Mosaicking

Due to the limited field of view of UAV-mounted thermal sensors, the acquired images exhibit significant spatial overlap and require mosaicking to generate a continuous thermal orthomosaic. In this study, a feature-based image registration method was adopted using the Speeded-Up Robust Features (SURF) algorithm, owing to its robustness to scale and rotation variations.

The SURF algorithm detects feature points based on the Hessian matrix, defined as [35]:

(1) $$H\left(f\left(x,y\right)\right)=\left[\begin{array}{cc} \frac{\partial^{2}f}{\partial^{2}x} & \frac{\partial^{2}f}{\partial x\partial y}\\ \frac{\partial^{2}f}{\partial x\partial y} & \frac{\partial^{2}f}{\partial^{2}y} \end{array}\right]$$

where *f*(*x*,*y*) denotes the image intensity at pixel location (*x*,*y*), and the second-order partial derivatives represent the local curvature of the image intensity in different directions. The determinant of the Hessian matrix is used to identify keypoints:

(2) $$Det(H)=\frac{\partial^{2}f}{\partial x^{2}}\frac{\partial^{2}f}{\partial y^{2}}-{\left(\frac{\partial^{2}f}{\partial x\partial y}\right)}^{2}$$

which reflects the intensity variation in the neighborhood and enables detection of salient feature points. In practice, the second-order derivatives are approximated using box filters for computational efficiency:

(3) $$Det(H)\approx D_{xx}D_{yy}-{\left(0.9D_{xy}\right)}^{2}$$

where *D_xx_*, *D_yy_*, and *D_xy_* denote the approximated second-order responses obtained via box filtering.

To improve efficiency and mitigate cumulative errors associated with conventional pairwise registration, a multi-frame mosaicking strategy was adopted. Feature points were first extracted from all images and matched across overlapping regions. Robust geometric transformations were then estimated using the MSAC algorithm, followed by global optimization to align all frames within a unified coordinate system. This strategy effectively reduces error propagation and improves spatial consistency in large-scale UAV datasets.

**Figure 6:**
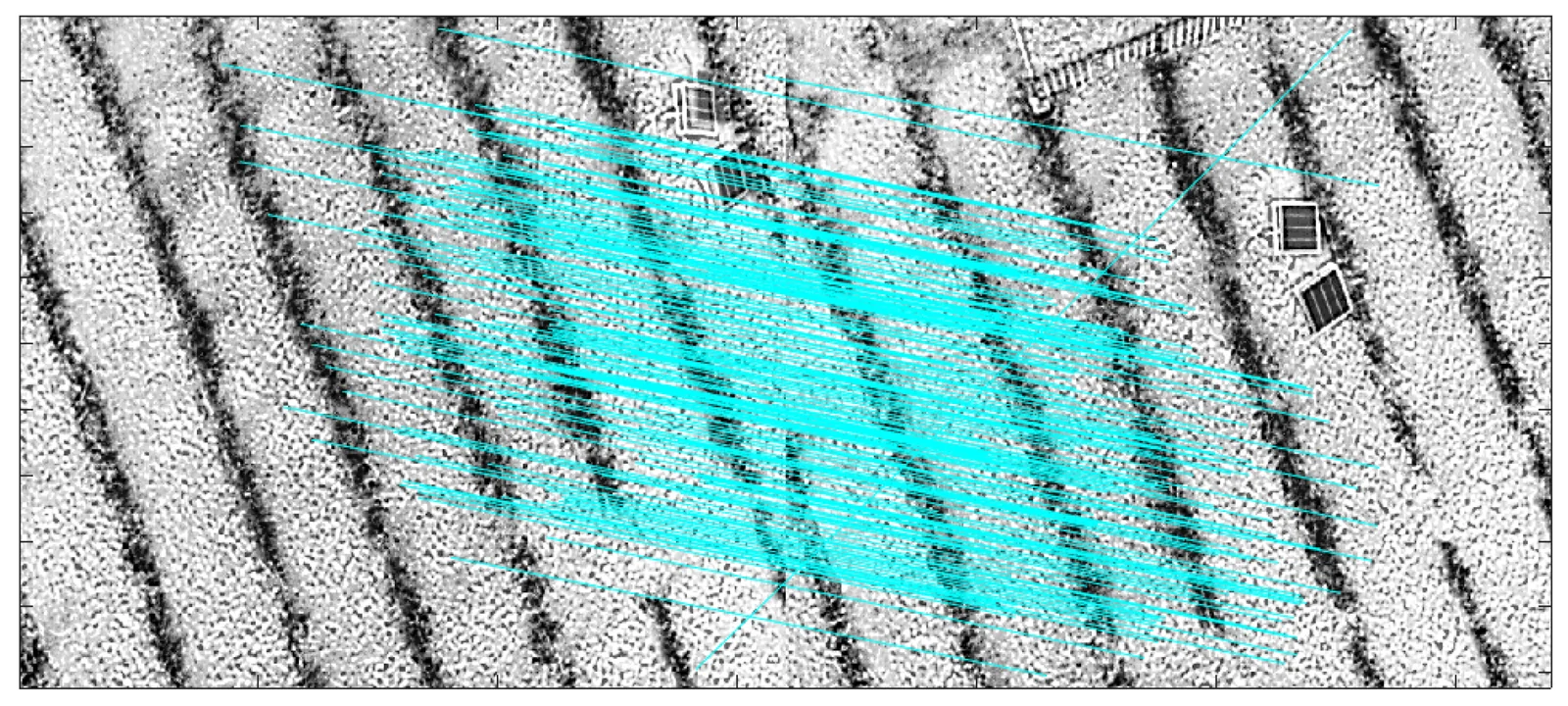
Feature matching results between overlapping UAV thermal images based on the SURF algorithm. The cyan lines indicate matched feature pairs between the overlapping thermal images.

Finally, feature correspondences were established between overlapping images based on the SURF descriptors, providing the basis for geometric transformation estimation. As shown in Figure 6, dense and reliable feature matches can be identified between adjacent frames, ensuring robust image registration. Subsequently, all images were projected onto a common reference plane and blended to generate a seamless thermal orthomosaic.

#### 2.2.2. Canopy Extraction and Background Removal

Accurate canopy temperature retrieval from thermal imagery requires effective separation of vegetation pixels from soil background, as mixed pixels can introduce significant bias in temperature estimation. In UAV-based thermal images, canopy and soil typically exhibit distinct radiometric characteristics, enabling segmentation based on pixel intensity differences [39].

In this study, a threshold-based segmentation approach was employed due to its computational efficiency and suitability for large-scale image processing. The thermal images were first converted into grayscale representations, where pixel intensity *G*(*x*,*y*) corresponds to the radiometric response at location (*x*,*y*). A binary mask *M*(*x*,*y*) was then constructed as:

(4) $$M(x,y)=\left\{\begin{array}{ll} 0, & G_{min}<G(x,y)<G_{max}\\ 1, & \mathrm{otherwise} \end{array}\right.$$

where *G_min_* and *G_max_* represent the lower and upper threshold values defining the canopy pixel range. Pixels within the threshold interval are classified as vegetation, while those outside are considered background.

Based on this mask, background removal was performed by:

(5) $$G_{c}(x,y)=G(x,y)\cdot (1-M(x,y))$$

where *G_c_*(*x*,*y*) denotes the processed image containing only canopy pixels. This operation effectively suppresses soil background by assigning zero values to non-canopy regions while preserving the original radiometric information of vegetation pixels.

To determine optimal threshold values, grayscale histograms of thermal images were analyzed. Typically, the histogram exhibits a bimodal distribution corresponding to canopy and soil regions. In this study, the threshold interval [*G_min_*, *G_max_*] was determined by combining histogram analysis with visual comparison between the RGB image and the corresponding thermal orthomosaic. The canopy-dominated gray-level range was identified from the main vegetation peak in the histogram, while soil/background pixels were excluded according to the secondary gray-level peak and the RGB reference image. Otsu’s method was not used because the objective was to determine a canopy grayscale interval rather than a single foreground–background threshold [39]. Instead, the threshold interval was optimized by adjusting *G_min_* and *G_max_* according to the grayscale histogram and visual inspection of the segmentation results, so that canopy pixels were retained while soil/background pixels were excluded. The effectiveness of the proposed method was evaluated by comparing pixel intensity distributions before and after segmentation. The disappearance of the secondary peak associated with soil pixels confirms successful background removal. However, no independently labeled ground-truth segmentation masks were available in this study. Therefore, quantitative segmentation metrics, such as pixel accuracy, precision, recall, and intersection over union, were not calculated. This is a limitation of the present work. Future studies should include manually annotated canopy masks or field-surveyed reference data to quantitatively validate canopy extraction accuracy.

**Figure 7:**
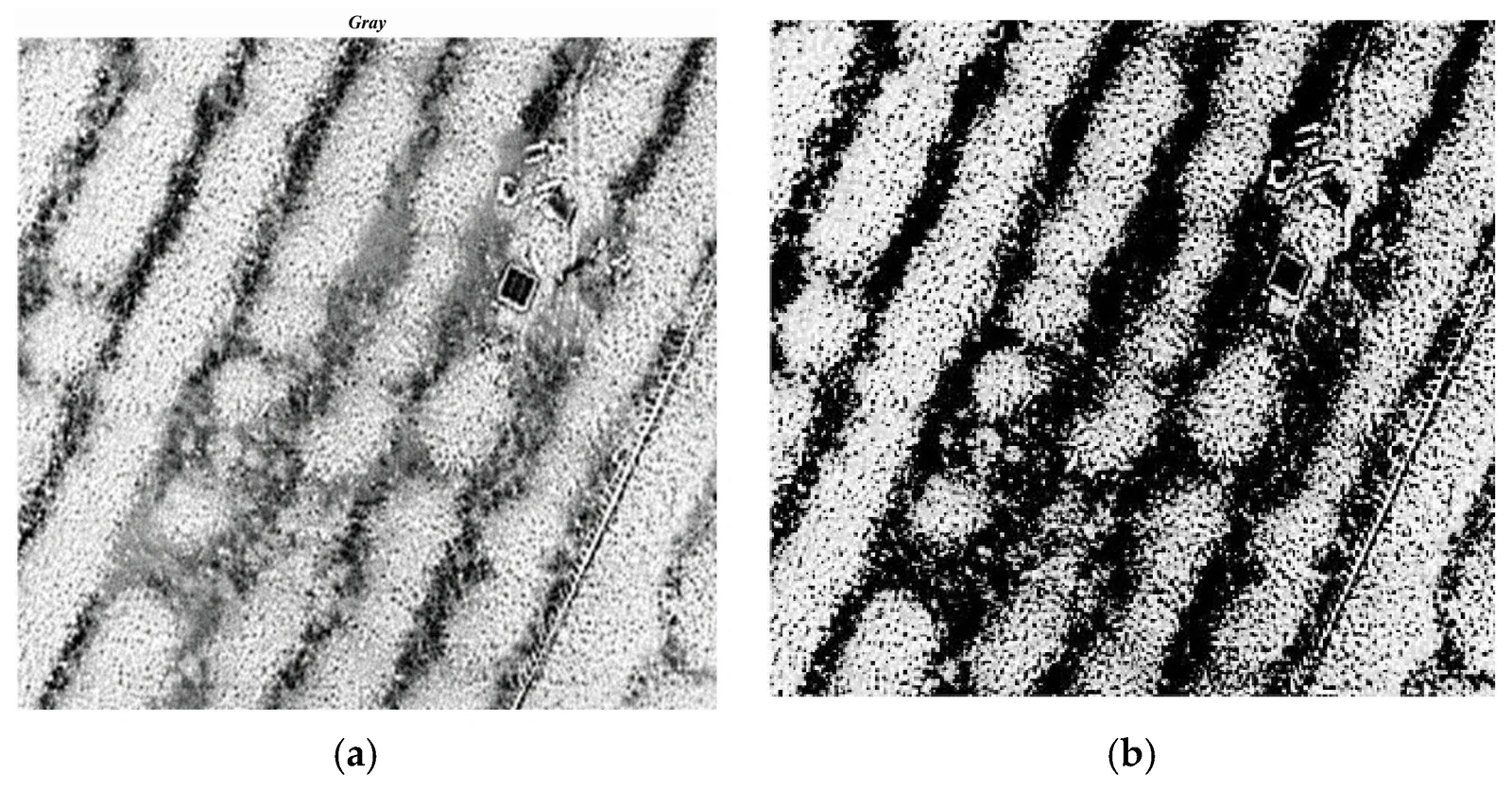
Canopy extraction and soil background removal from thermal imagery. (**a**) Original grayscale thermal image; (**b**) processed image after threshold-based segmentation, where soil background is suppressed and canopy pixels are retained.

As illustrated in Figure 7, the proposed method effectively removes soil background while retaining canopy structures. In addition, the disappearance of the secondary peak in the pixel intensity histogram after segmentation further confirms the effectiveness of the approach. This preprocessing step ensures that subsequent temperature retrieval is performed exclusively on canopy pixels, thereby improving the accuracy and reliability of canopy temperature estimation.

#### 2.2.3. Temperature Retrieval Model

To quantitatively retrieve canopy temperature from thermal imagery, a gray–temperature mapping model was established based on radiometric calibration. Although the thermal infrared camera can provide radiometric temperature information, the image mosaicking, geometric transformation, and canopy-background separation procedures in this study were performed on the processed thermal image matrix. After these operations, the retained canopy pixels were represented by grayscale values rather than directly by the original radiometric temperature output. Therefore, a gray–temperature conversion model was required to recover the physical temperature information of the extracted canopy pixels.

The grayscale value of a thermal infrared image reflects the digital response of the sensor to received infrared radiation. Under fixed camera settings, emissivity configuration, gain mode, and relatively stable environmental conditions, this digital response maintains a monotonic relationship with object temperature within a limited temperature range. Therefore, a functional relationship between grayscale value and actual temperature can be constructed under consistent imaging conditions [40]. In this study, explicit atmospheric correction and vegetation emissivity correction were not applied. Because the UAV flights were conducted at relatively low altitudes of 25 m, 40 m, and 60 m, the atmospheric path length was short, and the influence of atmospheric attenuation and path radiance was expected to be relatively limited at the UAV observation scale. The gray-temperature calibration model was established using synchronously collected ground-truth leaf temperature measurements, which partly compensated for systematic effects related to sensor response, emissivity setting, and residual atmospheric influence under the specific experimental conditions. Nevertheless, this model should be regarded as an empirical calibration model for the present UAV dataset rather than a universal physically based thermal inversion model.

In this study, because the canopy temperature range observed during the UAV flights was relatively narrow, the relationship between grayscale value *G* and temperature *T* was assumed to be approximately linear within the experimental temperature range and can be expressed as:

*T* = *kG* + *b* (6)

where *T* denotes the temperature (°C), *G* represents the grayscale value of the pixel, and *k* and *b* are the calibration coefficients determined through regression analysis.

To estimate the model parameters, a set of representative sample points was selected from multiple thermal images. For each sample point, the corresponding temperature value was obtained from the DJI Thermal Analysis Tool 2.1, while the grayscale value was extracted from the same image location in the processed image matrix. A total of 50 sample pairs were collected, among which 40 samples were used for model fitting and the remaining 10 samples were reserved for validation. The calibration dataset is summarized in Table 2, covering the temperature range observed in the study area. The 50 sample pairs covered the canopy temperature range from 29.0 °C to 43.4 °C. Since the gray–temperature model is a one-variable linear calibration model with only two regression coefficients, these samples were sufficient to establish the empirical conversion relationship under the fixed sensor configuration and experimental conditions of this study. It should be emphasized that the purpose of this calibration was not to develop a generalized temperature inversion model applicable to all environmental conditions, but to convert processed grayscale canopy pixels into temperature values for the present UAV thermal dataset.

The regression coefficients were estimated using the least squares method, yielding *k* = 0.109, *b* = 23.674. Thus, the final gray–temperature mapping model is expressed as:

*T* = 0.109*G* + 23.674 (7)

For validation, an independent dataset (Table 3) was used to evaluate the predictive accuracy of the model.

The comparison between predicted and measured temperatures indicates that the maximum absolute error is below 0.3 °C, with an average error of approximately 0.23 °C. These results demonstrate that the proposed linear model provides sufficient accuracy for canopy temperature retrieval under the given experimental conditions. Finally, the calibrated model was applied to all canopy pixels extracted in Section 2.2.2, enabling spatially distributed temperature estimation across the entire thermal orthomosaic. It should be noted that this gray–temperature model is an empirical calibration model rather than a universal radiative transfer model. Its applicability is limited to the sensor configuration, emissivity setting, gain mode, flight period, atmospheric conditions, and temperature range covered by the present experiment. When the camera type, gain mode, emissivity setting, flight altitude, weather condition, or crop canopy structure changes significantly, recalibration using ground temperature measurements or radiometric reference targets is required. Therefore, the proposed linear model is suitable for canopy temperature retrieval under the experimental conditions of this study, but its generalization to other environments should be further validated.

### 2.3. CWSI-Based Water Stress Estimation

Crop water stress was quantified using the Crop Water Stress Index (CWSI), which integrates canopy temperature with atmospheric conditions to characterize plant water status. The CWSI is defined as [41]:

(8) $$CWSI=\frac{\left(T_{c}-T_{a}\right)-{\left(T_{c}-T_{a}\right)}_{min}}{{\left(T_{c}-T_{a}\right)}_{max}-{\left(T_{c}-T_{a}\right)}_{min}}$$

where *T_c_* is the canopy temperature (°C) derived from UAV thermal imagery, and *T_a_* is the air temperature (°C) measured by the field meteorological station. (*T_c_* − *T_a_*)_min_ is the lower limit corresponding to low water-stress conditions, and (*T_c_* − *T_a_*)_max_ is the upper limit corresponding to the highest water-stress condition observed in the field experiment.

In this study, (*T_c_* − *T_a_*)_min_ and (*T_c_* − *T_a_*)_max_ were determined from field-observed canopy-air temperature differences under the experimental conditions. Specifically, (*T_c_* − *T_a_*)_min_ was obtained from the minimum observed *T_c_* − *T_a_* value among sampling points with relatively high soil water content and no obvious visual water-stress symptoms. In contrast, (*T_c_* − *T_a_*)_max_ was obtained from the maximum observed *T_c_* − *T_a_* value among sampling points with relatively low soil water content during the experimental period.

Meteorological data, including air temperature, relative humidity, wind speed, and net radiation, were synchronously recorded during UAV flights. The canopy temperature Tc was obtained from the processed thermal orthomosaic, ensuring that only vegetation pixels were considered in the analysis. The CWSI was computed at the plot scale by averaging canopy temperature within each experimental unit. Higher CWSI values indicate greater water stress, reflecting reduced transpiration and stomatal closure, whereas values close to zero correspond to well-irrigated or low-stress conditions. The derived CWSI values were subsequently used as the key input variable for irrigation decision-making, enabling a quantitative link between thermal remote sensing observations and water management strategies.

### 2.4. Irrigation Decision Model

In the methodology section, an irrigation decision model was developed to translate the remotely sensed crop water stress into quantitative irrigation requirements. The model establishes a functional relationship between the Crop Water Stress Index (CWSI) and soil water availability, and subsequently determines irrigation amount and duration.

#### 2.4.1. Soil Water Estimation Based on CWSI

CWSI is physically linked to crop transpiration and soil moisture conditions [42]. Under field conditions, a linear relationship between soil volumetric water content *θ* and CWSI can be assumed:

(9) $$\theta =\theta_{wp}+\left(\theta_{fc}-\theta_{wp}\right)(1-CWSI)$$

where *θ* is the soil volumetric water content (m^3^·m^−3^), *θ_wp_* is the wilting point, and *θ_fc_* is the field capacity. This formulation implies that increasing water stress (higher CWSI) corresponds to decreasing available soil moisture. It should be noted that this relationship was used as a simplified decision-support assumption in this study, rather than a fully validated soil moisture inversion model. Direct validation against synchronous in situ soil moisture measurements should be conducted in future work to further improve the quantitative accuracy of irrigation volume estimation.

#### 2.4.2. Irrigation Requirement Estimation

The irrigation water requirement per unit area *I* (mm) is defined as the amount of water needed to restore soil moisture from the current level *θ* to field capacity:

(10) $$I=\left(\theta_{fc}-\theta \right)\cdot Z_{r}$$

where *Z_r_* is the effective root zone depth (mm). Substituting Equation (9) into Equation (10), irrigation demand can be directly expressed as a function of CWSI:

(11) $$I=\left(\theta_{fc}-\theta_{wp}\right)\cdot CWSI\cdot Z_{r}$$

This formulation enables rapid estimation of irrigation requirements using remotely sensed canopy temperature data.

#### 2.4.3. Irrigation Scheduling Strategy

A threshold-based irrigation strategy was adopted to determine irrigation timing. Based on established agronomic practices, a CWSI threshold of 0.3 was used:

CWSI ≤ 0.3: no irrigation required; CWSI > 0.3: irrigation triggered.

The total irrigation volume *V* (m^3^) is then calculated as:

(12) V=I⋅A

where *A* is the field area (m^2^). The irrigation duration *t* (min) is determined based on the system flow rate *Q* (m^3^·min^−1^):

(13) $$t=\frac{V}{Q}$$

To account for system response delay, a short buffer time was added to ensure sufficient water supply during operation. The proposed irrigation decision model integrates UAV-derived canopy temperature, meteorological measurements, and soil parameters into a unified framework. All computations were implemented in a MATLAB R2020b enabling automated processing from image input to irrigation scheduling output. This model provides a direct linkage between thermal remote sensing observations and irrigation management, offering a practical and scalable solution for precision agriculture applications.

## 3. Results and Discussion

### 3.1. Performance of Thermal Image Mosaicking

**Figure 8:**
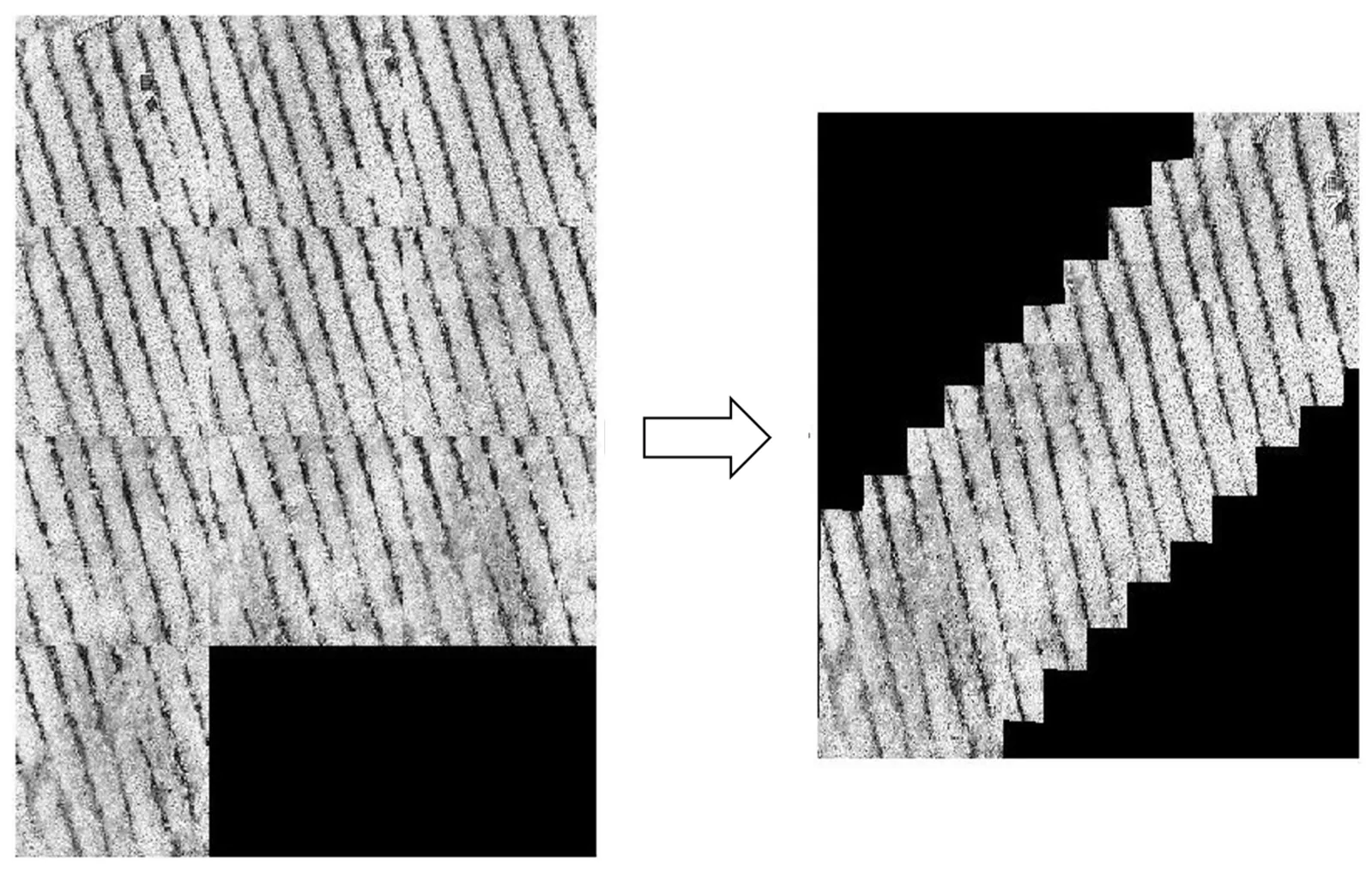
Thermal orthomosaic generated from multi-frame UAV images using the proposed SURF-based mosaicking method.

The mosaicking performance was evaluated using UAV-acquired thermal infrared images with high overlap. The resulting orthomosaic generated by the proposed SURF-based multi-frame mosaicking method is shown in Figure 8. As observed, the mosaic exhibits good spatial continuity without visible seams or obvious geometric distortions. Crop rows and field boundaries are consistently aligned across image edges, indicating accurate geometric registration. The robustness of the method can be attributed to SURF-based feature matching and MSAC-based outlier rejection, which ensure stable transformation estimation and reduce cumulative alignment errors during multi-frame mosaicking. The generated orthomosaic provides a consistent spatial basis for subsequent canopy extraction and temperature retrieval.

To further evaluate the registration performance of the proposed feature-based mosaicking strategy, SIFT-, ORB-, and SURF-based registration methods were compared using the same UAV thermal infrared image sequence. Nine adjacent image pairs were used for evaluation, and all three methods were combined with MSAC-based robust transformation estimation. The evaluation metrics included the mean number of matched feature pairs, inlier ratio, mean reprojection error, and processing time.

As shown in Table 4, SURF + MSAC achieved the largest number of matched feature pairs, with a mean value of 81.78, followed by SIFT + MSAC and ORB + MSAC. In terms of geometric accuracy, SURF + MSAC produced the lowest mean reprojection error of 0.688 pixels, which was slightly lower than those of SIFT + MSAC and ORB + MSAC. ORB + MSAC achieved the highest inlier ratio and the shortest processing time, indicating good computational efficiency. However, it produced fewer matched pairs and a slightly higher reprojection error. Although ORB + MSAC achieved the highest inlier ratio and the shortest processing time, its mean number of matched feature pairs was lower than that of SURF + MSAC, and its mean reprojection error was slightly higher. For UAV thermal infrared image mosaicking, the inlier ratio alone is not sufficient to evaluate registration performance. A small number of highly consistent matches may produce a high inlier ratio, but may not provide enough spatial constraints for stable multi-frame mosaicking. In contrast, a larger number of reliable matched pairs can improve the robustness of geometric transformation estimation, especially in thermal infrared images with weak texture and low contrast. Therefore, SURF + MSAC was selected in this study because it produced the largest number of matched feature pairs and the lowest mean reprojection error, while maintaining acceptable processing time. This tradeoff indicates that SURF + MSAC provided a more suitable balance between feature abundance, geometric accuracy, and computational efficiency for the UAV thermal infrared image sequence used in this study.

### 3.2. Accuracy of Canopy Extraction and Background Removal

**Figure 9:**
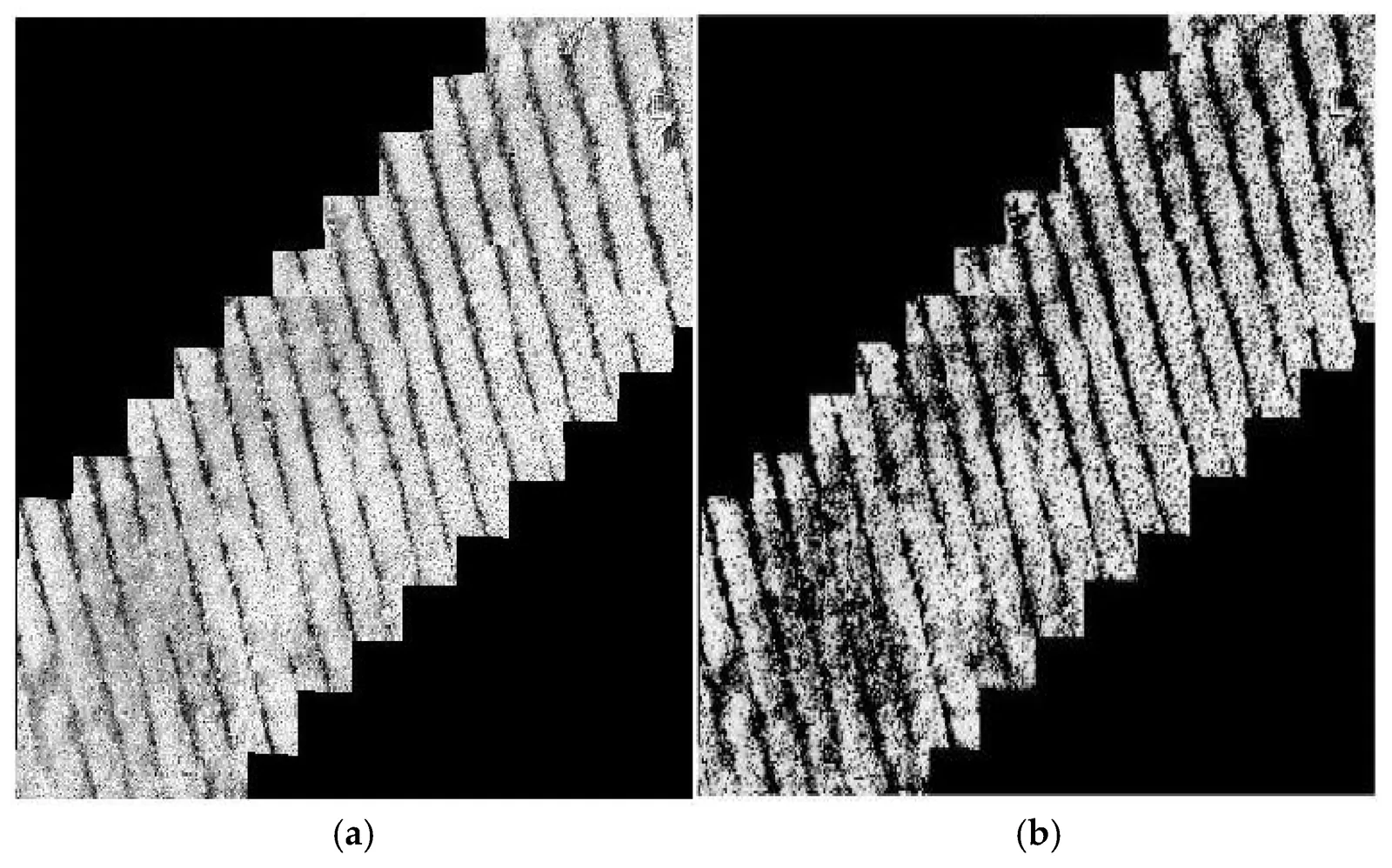
Image processing results: (**a**) Infrared thermal imaging mosaic; (**b**) Soil Background Removal Map.

**Figure 10:**
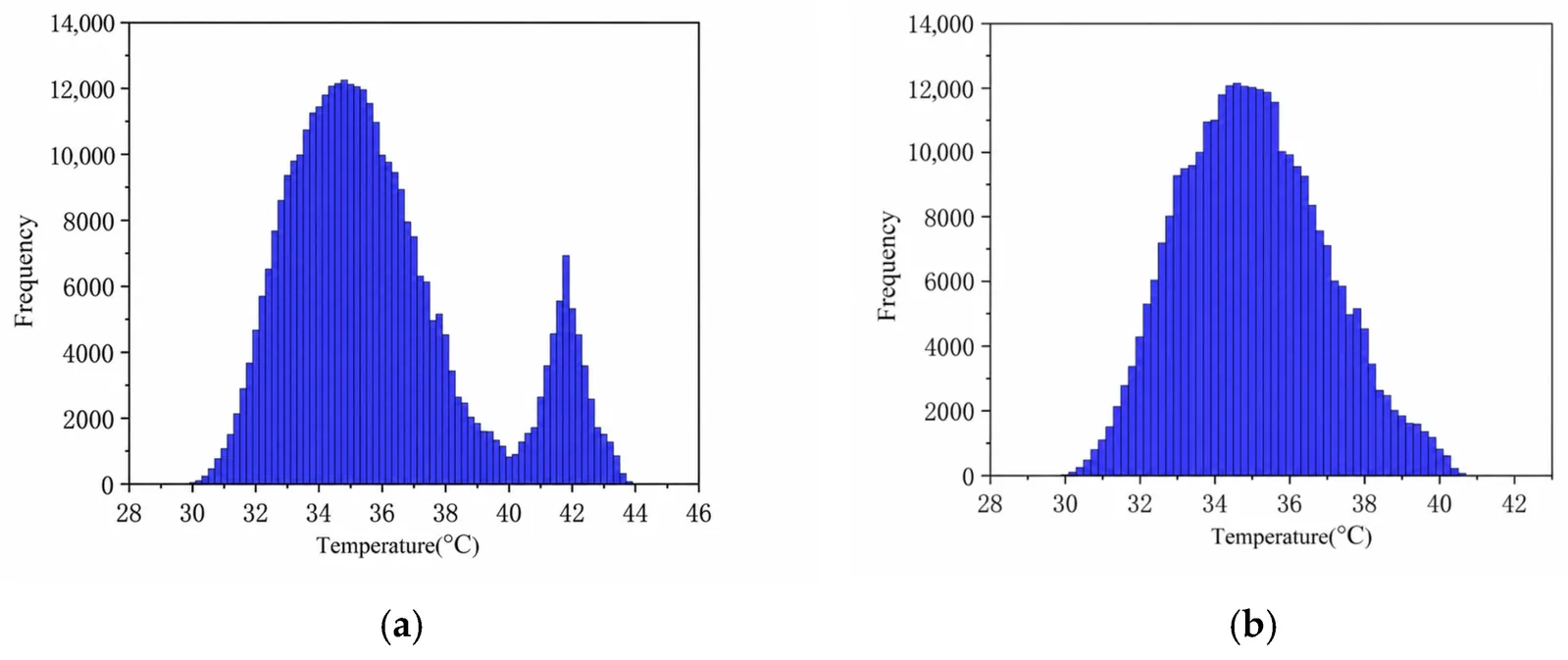
Histogram of infrared thermal image temperature: (**a**) Temperature histogram before removing soil background; (**b**) Temperature histogram after removing soil background.

The performance of the proposed image processing pipeline, including multi-frame mosaicking and soil background removal, is illustrated in Figure 9 and Figure 10. Figure 9a shows the mosaicked infrared thermal image, where overlapping UAV frames are accurately aligned without visible seams, demonstrating high geometric consistency. Figure 9b presents the same image after soil background removal, with non-canopy pixels effectively suppressed while retaining canopy structures.

The corresponding pixel intensity histograms, shown in Figure 10, further confirm the effectiveness of the background removal. Prior to processing, the histogram exhibits a bimodal distribution, where the first peak represents canopy pixels with lower temperatures and the second peak corresponds to soil pixels with higher temperatures. After applying the segmentation algorithm, the secondary peak is eliminated, resulting in a unimodal distribution dominated by canopy pixels. This confirms that the soil background has been largely removed, ensuring that subsequent temperature retrieval is performed exclusively on canopy pixels, which improves the accuracy and reliability of canopy temperature estimation.

### 3.3. Temperature Retrieval Performance

Canopy temperature across the study area was quantitatively retrieved using the gray–temperature calibration model described in Section 2.2.3. The linear relationship between pixel intensity and physical temperature was applied to all canopy pixels extracted from the thermal orthomosaic, generating a spatially continuous temperature map. To address the consistency between the predicted values and the regression equation, all predicted temperatures in the validation table were recalculated according to the established gray-temperature regression equation. The maximum absolute error observed was 0.285 °C. To provide a more comprehensive evaluation, standard statistical indicators, including mean absolute error (MAE), root mean square error (RMSE), Bias, and mean absolute percentage error (MAPE), were further calculated based on the independent validation dataset.

**Figure 11:**
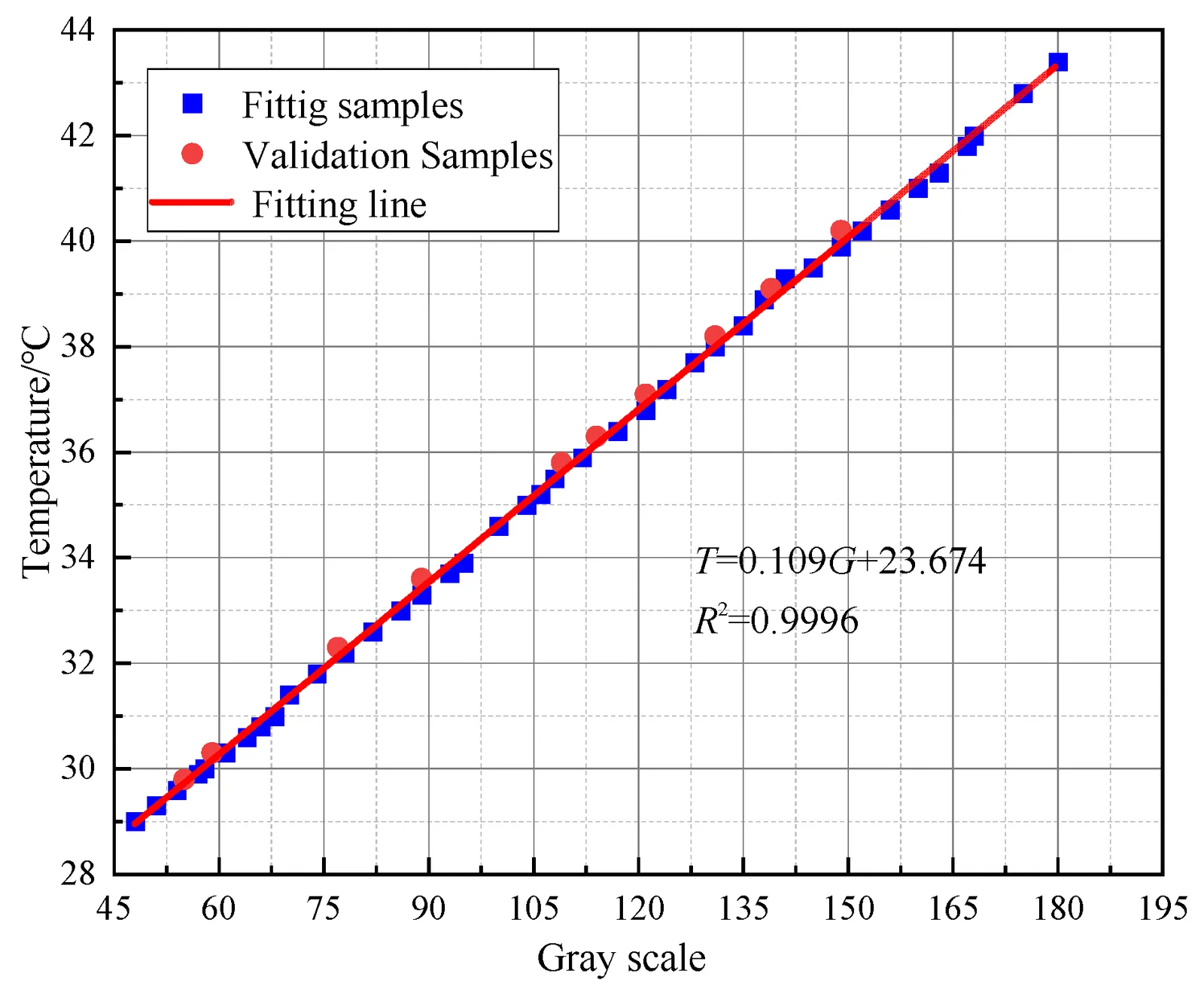
Linear regression between grayscale value and temperature.

Figure 11 presents the linear regression between grayscale value and measured temperature for the calibration dataset, illustrating the gray-temperature conversion relationship used in this study. Correspondingly, Table 5 lists the corrected validation results, including grayscale values, predicted temperatures recalculated from the regression equation, measured temperatures, and absolute errors. This approach ensures that temperature retrieval is confined exclusively to vegetation pixels, effectively minimizing bias from soil background, and provides a solid foundation for subsequent crop water stress analysis.

The evaluation metrics were calculated as follows:

(14) $$\mathrm{MAE}\;=\frac{1}{n}\sum_{i=1}^{n}\;\left|T_{\mathrm{pred},i}-T_{\mathrm{obs},i}\right|$$

(15) $$\mathrm{RMSE}=\sqrt{\frac{1}{n}\sum_{i=1}^{n}\;{\left(T_{\mathrm{pred},i}-T_{\mathrm{obs},i}\right)}^{2}}$$

(16) $$\mathrm{Bias}=\frac{1}{n}\sum_{i=1}^{n}\;\left(T_{\mathrm{pred},i}-T_{\mathrm{obs},i}\right)$$

(17) $$\mathrm{MAPE}=\frac{100\%}{n}\sum_{i=1}^{n}\;\left|\frac{T_{\mathrm{pred},i}-T_{\mathrm{obs},i}}{T_{\mathrm{obs},i}}\right|$$

where *T_pred_*,*_i_* is the predicted temperature, *T_obs_*,*_i_* is the observed temperature, and n is the number of validation samples.

The calculated evaluation results are summarized in Table 6. The MAE and RMSE were 0.227 °C and 0.231 °C, respectively, indicating that the prediction error of the gray–temperature calibration model was low. The Bias was −0.227 °C, suggesting a slight underestimation of canopy temperature. The MAPE was 0.641%, further confirming that the linear calibration model provided sufficient accuracy for canopy temperature retrieval under the experimental conditions.

### 3.4. Irrigation Decision Results

**Figure 12:**
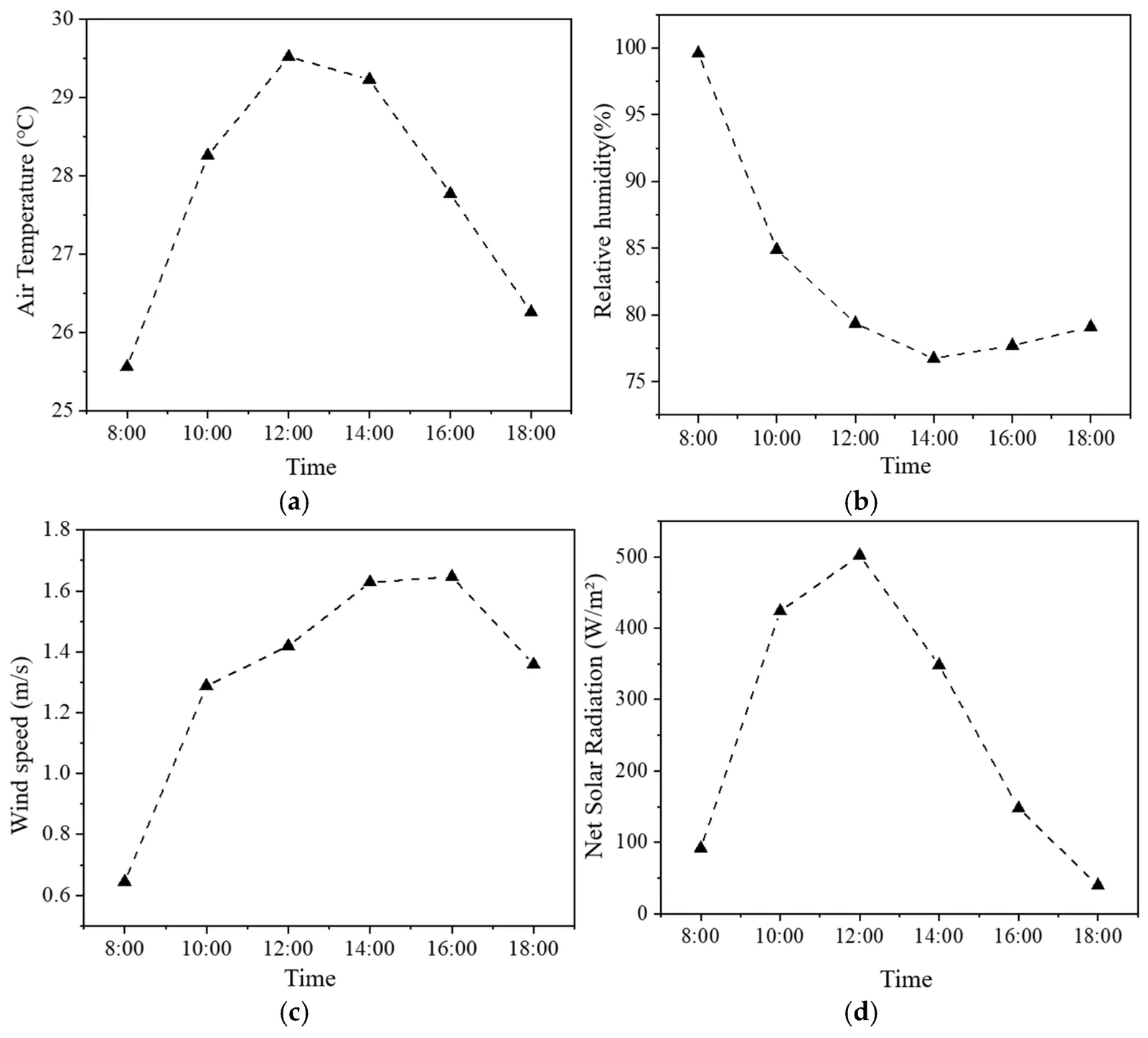
Related meteorological data. (**a**) Air temperature. (**b**) Relative humidity. (**c**) Wind speed. (**d**) Net Solar Radiation.

UAV-derived canopy temperature and the corresponding Crop Water Stress Index (CWSI) were used to estimate crop water stress and determine irrigation requirements across the experimental tea plantation. Meteorological data including air temperature, relative humidity, wind speed, and net solar radiation were recorded synchronously with UAV flights to ensure temporal consistency, as shown in Figure 12.

**Figure 13:**
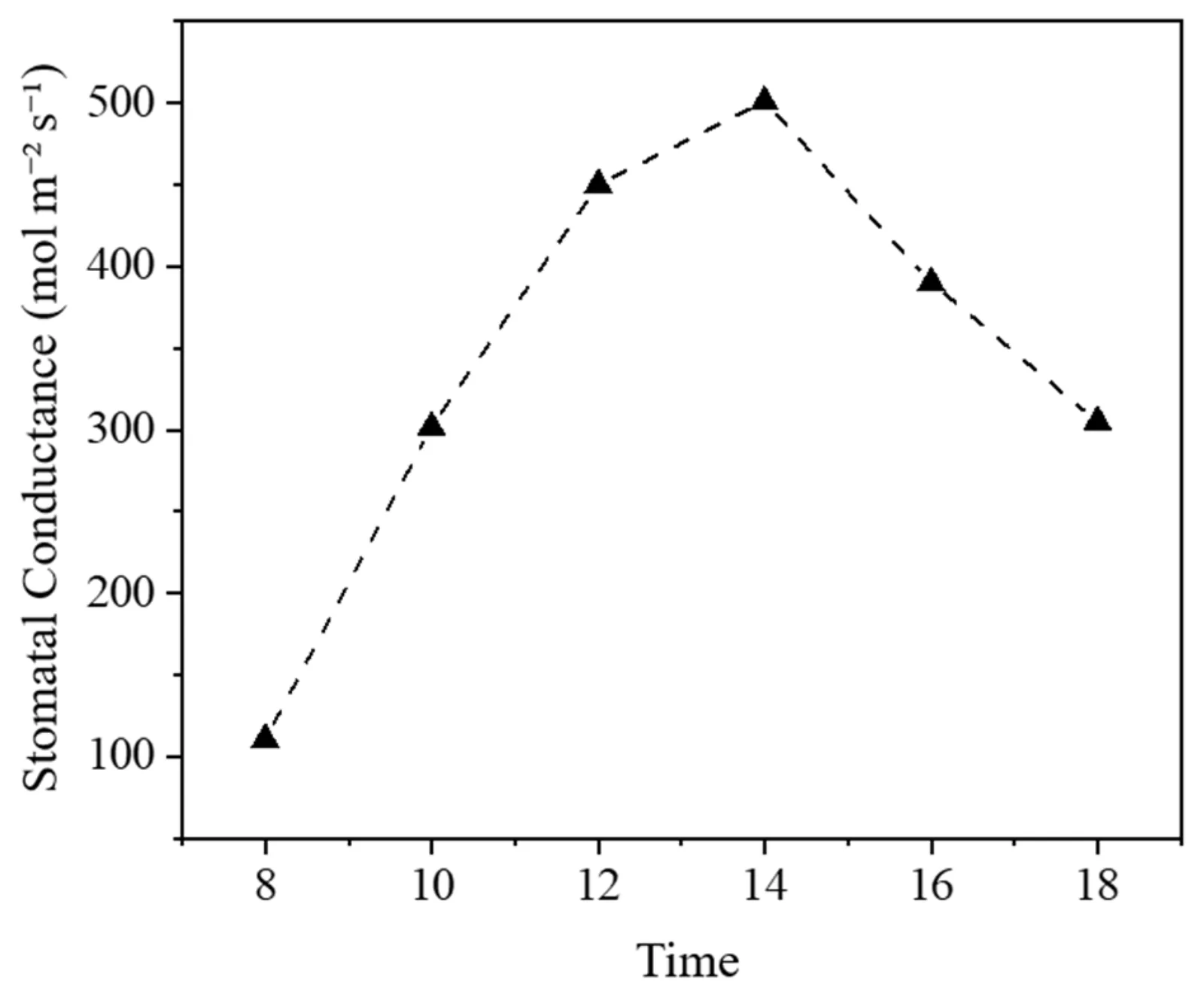
Crop stomatal conductance data.

Stomatal conductance (*Gs*), representing the degree of stomatal opening and directly reflecting plant transpiration, was measured at 20 representative sampling points using a LI-6400 portable photosynthesis system. For each sampling point, the mean of three to four representative leaves was used as the observed value shown in Figure 13. Higher CWSI values correspond to increased water stress, reduced transpiration, and lower stomatal opening. Therefore, *Gs* was used as an independent validation metric for water stress estimation and irrigation predictions. Predicted CWSI and irrigation values were compared against measured *Gs* using linear regression. The coefficient of determination *R*^2^ was calculated to quantify the correlation, defined as:

(18) $$R^{2}=1-\frac{\sum_{i=1}^{n}{\left(y_{i}-{\hat{y}}_{i}\right)}^{2}}{\sum_{i=1}^{n}{\left(y_{i}-\bar{y}\right)}^{2}}$$

where $y_{i}$ is the *i*-th measured value, ${\hat{y}}_{i}$ is the *i*-th predicted value, $\bar{y}$ is the mean of the measured values, and *n* is the number of samples.

Using the CWSI-based model, soil volumetric water content in the effective root zone was estimated, and irrigation requirements were calculated using a threshold strategy with CWSI greater than 0.3. The accuracy of the system was evaluated by correlating predicted CWSI and irrigation volumes with observed *Gs* values. Table 7 shows that CWSI exhibits a strong negative correlation with stomatal conductance at all flight times, with the highest correlation at 13:00 coinciding with peak plant water stress. Similarly, predicted irrigation volume per unit area shows strong correspondence with *Gs* as presented in Table 8, achieving *R*^2^ above 0.9 at midday. These results indicate that UAV-based thermal observations can reliably capture spatiotemporal patterns of crop water stress and provide a solid basis for precise irrigation scheduling.

## 4. Discussion

### 4.1. Canopy Temperature Retrieval from UAV Thermal Infrared Imagery

In this study, a UAV-based thermal infrared inversion method was developed for tea canopy temperature retrieval. The gray–temperature calibration model achieved high accuracy, with a maximum absolute error of less than 0.3 °C between predicted and measured temperatures. Previous studies have shown that UAV-derived thermal products need to be validated against ground measurements, and that UAV thermal imagery can provide higher spatial resolution and stronger agreement with in situ temperature than satellite thermal products in agricultural areas [23]. In addition, calibration studies on uncooled thermal infrared cameras have demonstrated that radiometric correction can substantially reduce measurement bias and vignette effects, with RMSE decreasing from 6.219 to 0.815 °C and from 3.438 to 1.013 °C in two thermal cameras after calibration [24]. Compared with these studies, the present method uses a simpler field-based gray–temperature calibration relationship to retrieve canopy temperature. This approach is easier to implement in tea plantation experiments, although it remains dependent on sensor settings, flight conditions, and environmental background.

Background removal was a key step for improving canopy temperature retrieval. After soil background removal, the coefficient of determination between image-derived and ground-measured canopy temperature increased from 0.7673 to 0.9355. This result indicates that the original thermal image contained mixed information from tea canopy, bare soil, shaded soil, and canopy edges. Previous studies combining thermal and visible imagery have shown that identifying leaf area and separating sunlit and shaded canopy components can improve thermal indices and their relationship with stomatal conductance [37]. Automatic canopy temperature extraction methods have also been shown to reduce operator subjectivity and image-processing time, supporting the use of thermography for plant water-status assessment and irrigation scheduling [38]. Compared with these studies, the present study directly quantified the improvement caused by background removal in UAV thermal orthomosaics of tea plantations. This is important because row-structured tea canopies and inter-row soil easily produce mixed pixels. Therefore, background removal is not only an image-processing step, but also a necessary procedure for obtaining physiologically meaningful canopy temperature.

Flight altitude also affected the interpretation of UAV-derived canopy temperature. Although thermal images were acquired at 25 m, 40 m, and 60 m, the main quantitative irrigation decision analysis was based on the 25 m dataset. Previous tea plantation research compared UAV thermal images acquired at 25 m, 40 m, and 60 m at 09:00, 11:00, and 13:00, and found that canopy temperature retrieval varied with both flight height and acquisition time; the 60 m orthomosaic at 11:00 provided the best relationship under its specific experimental conditions [43]. Therefore, the optimal flight altitude should not be regarded as fixed. In the present study, 25 m was selected mainly because it provided clearer tea canopy boundaries and lower mixed-pixel interference, which were beneficial for canopy-background separation and subsequent CWSI-based irrigation estimation. However, the influence of flight altitude on temperature retrieval and irrigation decision accuracy should be further quantified using synchronous ground measurements at multiple altitudes.

### 4.2. CWSI-Based Irrigation Decision-Making and Application Prospects

In this study, the UAV-derived CWSI showed a clear negative relationship with stomatal conductance, indicating that the retrieved canopy temperature reflected the physiological water status of tea plants. Similar relationships between CWSI and plant water-status indicators have been reported in wheat, potato, and orchard crops. For example, CWSI was linearly related to available root-zone water and grain yield in wheat, and a CWSI threshold of 0.26 was suggested for improving irrigation timing [7]. In potato, CWSI derived from multi-year ground and aerial thermal images was highly correlated with stomatal conductance, with *R*^2^ values ranging from 0.64 to 0.99 [8]. In nectarine and peach orchards, adaptive CWSI based on pure canopy extraction showed stronger relationships with stem water potential and stomatal conductance than conventional CWSI [9]. Compared with these studies, the present study extends CWSI-based water-stress assessment to tea plantations and verifies its reliability using stomatal conductance under a row-structured canopy condition.

More importantly, this study further connects CWSI-based water-stress diagnosis with irrigation requirement estimation. Previous studies have mainly used CWSI to identify water deficit, compare irrigation treatments, or map spatial variability of crop water status [7,8,9]. In contrast, the present study uses UAV-derived canopy temperature to calculate CWSI, estimate soil water status, and determine the irrigation requirement per unit area. This provides a more direct link between thermal remote-sensing information and irrigation management. A related UAV thermal study predicted soil moisture using canopy temperature and machine-learning models, providing data support for irrigation management [13], while recent *ET*_c_ estimation work further showed that CWSI and UAV-derived information can be converted into water-use variables [22]. Compared with these approaches, the proposed method uses a shorter and more interpretable route from canopy temperature retrieval to irrigation amount estimation, which is suitable for rapid irrigation decision-making in tea plantations.

However, the proposed method is still at the decision-support stage rather than a complete closed-loop irrigation control system. Smart irrigation systems require decisions on when, where, and how much water to apply, and closed-loop control generally depends on the integration of plant-, soil-, and weather-based monitoring information [3]. In this study, UAV thermal imagery provides spatially continuous plant water-stress information and irrigation amount estimation, but the results still need to be coupled with soil moisture sensors, meteorological data, and automatic irrigation equipment for field-scale implementation. Future work should therefore test the proposed method across different seasons, tea cultivars, and meteorological conditions, and evaluate its performance in an automatic irrigation control system. In addition, the proposed UAV thermal framework may provide useful input variables for future ML/AI-assisted irrigation systems. By integrating canopy temperature, CWSI, soil moisture sensor data, meteorological information, and irrigation operation records, data-driven models could be further developed to predict soil moisture dynamics and optimize irrigation timing and irrigation amount under variable field conditions. Therefore, although the present study mainly establishes an interpretable CWSI-based irrigation decision framework, it can serve as a field-scale sensing and decision-support basis for future intelligent irrigation models.

## 5. Conclusions

This study presents an integrated framework for UAV-based thermal infrared monitoring and irrigation decision-making in a tea plantation. Canopy temperature was quantitatively retrieved from high-resolution thermal imagery using a gray–temperature calibration model, and subsequent Crop Water Stress Index (CWSI) calculations enabled the assessment of crop water status. The combination of multi-frame image mosaicking, canopy extraction with soil background removal, and linear temperature retrieval demonstrated high accuracy, with maximum absolute error below 0.3 °C and strong correlation (*R*^2^ > 0.999) between measured and predicted values.

Meteorological and physiological data were incorporated to validate model predictions. Stomatal conductance (*Gs*) measurements revealed strong negative correlations with both CWSI and predicted irrigation volumes, particularly at peak transpiration times, confirming the reliability of the UAV-based water stress assessment. The CWSI-derived soil water estimation model, coupled with threshold-based irrigation decision criteria (CWSI > 0.3), enabled precise calculation of irrigation requirements and duration. Linear regression analysis indicated that the proposed framework consistently captured the dynamic relationships between canopy temperature, plant water stress, and irrigation demand, with *R*^2^ values exceeding 0.9 for midday measurements. Overall, the proposed methodology provides a robust, high-resolution, and operationally feasible approach for precision irrigation management. Key conclusions include:

1. UAV-based thermal imaging, combined with accurate radiometric calibration and soil background suppression, provides reliable canopy temperature data for large-scale agricultural fields.
2. The CWSI-based irrigation decision model quantitatively links remotely sensed canopy temperature with root-zone soil moisture and irrigation requirements, demonstrating strong agreement with independent physiological measurements.
3. The framework demonstrates potential for scaling to diverse crops and field conditions, offering a unified methodology to bridge remote sensing observations with actionable irrigation strategies, ultimately enhancing water use efficiency and supporting sustainable agricultural management.

These findings provide both methodological innovation and practical guidance for UAV-based precision irrigation, highlighting the feasibility of combining high-resolution thermal monitoring, physiological validation, and automated decision-making for effective crop water management.

## Figures and Tables

**Table 1:** Thermal camera and RGB camera parameters.

Specification	Infrared Thermal Imaging Camera (Zenmuse XT2)	RGB Camera (Phantom 4 RTK)
Payload/Takeoff Weight	2.7 kg (Payload)/9 kg (Takeoff weight)	1391 g (Takeoff weight)
Scene Range	High Gain: −25 to 150 °C Low Gain: −40 to 550 °C	Temperature range: 0 to 40 °C
Flight Time	55 min	30 min
Sensor Type	Uncooled Vanadium Oxide (VOx) Microbolometer	CMOS
Image Resolution	640 × 512	4864 × 3648
Pixel Pitch	12 µm	—
Spectral Band/Wavelength	8–14 µm/7.5–13.5 µm	—
Sensitivity (NETD)	<50 mk@f/1.0	—
Digital Zoom/Distance	1×, 2×, 4×, 8×	350 mm
Exposure Mode	Auto, Manual	Auto, Manual
Photo Formats	JPEG, TIFF, R-JPEG(16-bit)	JPEG
Storage	MicroSD card	MicroSD card
Operating Frequency	—	2.400 GHz to 5.850 GHz
Transmission Power	—	5.8 GHz

**Table 2:** Calibration dataset for gray–temperature model fitting.

Grayscale Value	Temperature Value/°C	Grayscale Value	Temperature Value/°C	Grayscale Value	Temperature Value/°C
48	29.0	86	33.0	131	38.0
51	29.3	89	33.3	135	38.4
54	29.6	93	33.7	138	38.9
57	29.9	95	33.9	141	39.3
58	30.0	100	34.6	145	39.5
61	30.3	104	35.0	149	39.9
64	30.6	106	35.2	152	40.2
66	30.8	108	35.5	156	40.6
68	31.0	112	35.9	160	41.0
70	31.4	117	36.4	163	41.3
74	31.8	121	36.8	167	41.8
78	32.2	124	37.2	168	42.0
82	32.6	128	37.7	175	42.8
				180	43.4

**Table 3:** Validation dataset for gray–temperature model.

Grayscale Value	Temperature Value/°C	Grayscale Value	Temperature Value/°C
55	29.8	114	36.3
59	30.3	121	37.1
77	32.3	131	38.2
89	33.6	139	39.1
109	35.8	149	40.2

**Table 4:** Comparison of SIFT-, ORB-, and SURF-based registration methods for UAV thermal infrared image mosaicking.

Method	Mean Matched Pairs	Mean Inlier Ratio/%	Mean Reprojection Error/Pixel	Mean Processing Time/s
SIFT + MSAC	76.33	58.14	0.689	0.082
ORB + MSAC	63.00	72.57	0.713	0.026
SURF + MSAC	81.78	42.86	0.688	0.072

**Table 5:** Validation of canopy temperature predictions: comparison between predicted values, measured temperatures, and absolute errors (°C).

Grayscale Value	Temperature Predicted Value/°C	Measured Temperature/°C	Absolute Error/°C
55	29.669	29.8	0.131
59	30.105	30.3	0.195
77	32.067	32.3	0.233
89	33.375	33.6	0.225
109	35.555	35.8	0.245
114	36.100	36.3	0.200
121	36.863	37.1	0.237
131	37.953	38.2	0.247
139	38.825	39.1	0.275
149	39.915	40.2	0.285

**Table 6:** Evaluation metrics of the gray–temperature calibration model.

Metric	Value
MAE/°C	0.227
RMSE/°C	0.231
Bias/°C	−0.227
MAPE/%	0.641
Maximum absolute error/°C	0.285

**Table 7:** Correlation between CWSI and *Gs*.

Flight Time/h	Flight Altitude/m	Model Equation	*R* ^2^
9:00	25	*y* = −730.4*x* + 711.26	0.7905
11:00	25	*y* = −1506.9*x* + 988.18	0.7867
13:00	25	*y* = −735.57*x* + 674.06	0.9064

**Table 8:** Correlation between crop irrigation amount and *Gs*.

Flight Time/h	Flight Altitude/m	Model Equation	*R* ^2^
9:00	25	*y* = −13,646*x* + 472.88	0.8009
11:00	25	*y* = −23,860*x* + 509.78	0.8542
13:00	25	*y* = −13,374*x* + 453.39	0.9046

## Data Availability

The data presented in this study are available on request from the corresponding author.
